# Utility of Ultrasound Imaging Features in Diagnosis of Breast Cancer

**DOI:** 10.7759/cureus.37691

**Published:** 2023-04-17

**Authors:** Sultan A Alshoabi, Amal A Alareqi, Fahad H Alhazmi, Abdulaziz A Qurashi, Awatif M Omer, Abdullgabbar M Hamid

**Affiliations:** 1 Diagnostic Radiology Technology, Taibah University College of Applied Medical Sciences, Almadinah Almunawwarah, SAU; 2 Radiology, University of Medical and Applied Science, Sana’a, YEM; 3 Radiology, National Cancer Control Foundation (NCCF), Sana’a, YEM; 4 Radiology, Rush University Medical Center, Chicago, USA

**Keywords:** lymphadenopathy, tissue distortion, hypo-echogenicity of the mass, spiculated margins of the mass, irregular shape of the mass, specificity of ultrasound imaging features, breast cancer

## Abstract

Background

Currently, breast cancer (BC) is considered one of the most prevalent cancer worldwide in women and represents a global health challenge. Early diagnosis is the keystone in the management of BC patients. This study aims to assess the utility of ultrasonography (US) features of malignancy in the diagnosis of BC.

Methods

This retrospective cross-sectional study involved the electronic records of 326 female patients who were diagnosed with BC. A cross-tabulation test was performed to identify the association between the presence of each US feature (yes/no), and the final US diagnosis (benign/malignant). The strength of association of each feature was measured using the odds ratio (OR) which was assumed to be significant when > 1, with a 95% confidence interval (CI).

Results

The mean age of the female patients involved in this study was 45.36 ±12.16 years old (range, 17-90 years). Cross-tabulation test showed a significant association between the malignancy tumor and the irregular shape of the lesion (p < 0.001, OR=7.162, CI 2.726-18.814), non-circumscribed margins (p < 0.001, OR = 9.031, CI 3.200-25.489), tissue distortion (p < 0.001, OR = 18.095, CI 5.944-55.091), and the lymph node enlargement (p < 0.001, OR = 5.705, CI 2.332-13.960).

Conclusion

US imaging features of malignancy have a high sensitivity and positive predictive value for detection of the BC. However, the specificity of breast US imaging features is much lower because of the overlapping features in benign and malignant breast lesions. Breast lesions with an irregular shape, not circumscribed irregular or spiculated margins, hypo-echogenicity, tissue distortion, and those with lymphadenopathy have the highest likelihood of malignancy despite the low specificity. US is a highly valuable, safe, and affordable imaging modality with high diagnostic accuracy for BC.

## Introduction

Breast cancer (BC) is currently considered one of the most commonly diagnosed cancers worldwide and represents a global health challenge [1]. In 2020, World Health Organization (WHO) report showed that BC was responsible for nearly 685,000 female deaths worldwide [2]. Ultrasonography (US) is a widely available, radiation-free, non-invasive, and effective primary tool for the early detection of BC, with high sensitivity and specificity [3]. The US can distinguish breast cysts, probably benign, and suspicious masses. On the other hand, it is less affected by breast density compared with Mammography (MG). It is indicated as the sole and preferred imaging modality to evaluate focal signs and symptoms of breast lesions in females younger than 30 years old [4]. Compared to MG, the US can detect lesions smaller than 2 cm even in patients with high breast density [5]. The US features to describe the breast lesion as the following: 1) shape (round/oval, lobular, or irregular), 2) margins (circumscribed or not circumscribed, micro lobulated, angular, and speculated), 3) echogenic pattern (hyperechoic, hypoechoic, isoechoic, or mixed echogenicity), 4) orientation (parallel or antiparallel), 5) boundary (abrupt, or halo), and 6) posterior acoustic features (enhancement, shadowing, mixed, or none) [6]. US features of malignancy include irregular, spiculated, or angular margins, taller than wider orientation, microcalcification, and posterior acoustic shadowing [7,8].

As per the literature, most of the studies investigated the sensitivity of US either alone or as a supplemental technique to MG. In clinical practice, the US is a widely used diagnostic method for BC. We suspect that it is a highly accurate imaging modality for BC. This study aims to elucidate the diagnostic accuracy of each feature of malignancy in US imaging as a widely used primary tool for BC imaging.

## Materials and methods

Study design

**Figure 1:**
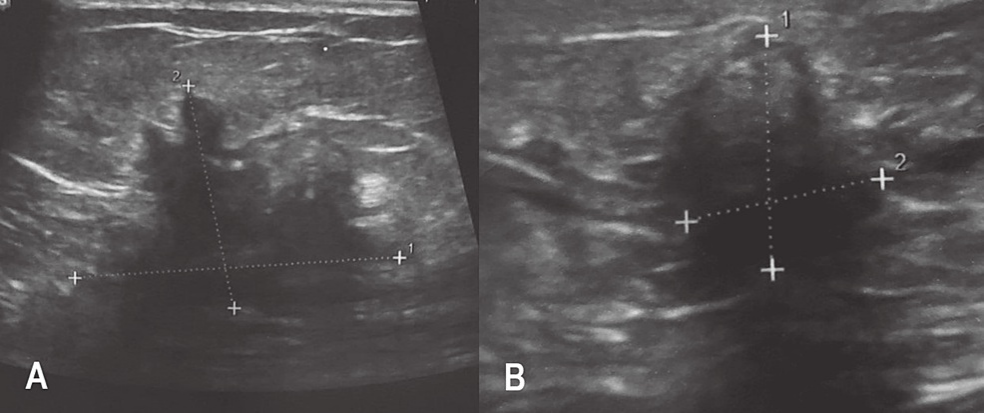
ultrasound images of two different patients showing a) irregular shape, partially ill-defined spiculated margin, hypoechogenic breast lesion with posterior acoustic shadowing, b) irregular shape, partially ill-defined spiculated margin, hypoechogenic breast lesion with posterior acoustic shadowing. Both lesions were malignant lesions as proved by histopathology examinations.

In this retrospective study, 336 patients with histopathologically confirmed BC were involved. The study was undertaken at the Life Center for Cancer Early Detection of the National Cancer Control Foundation (NCCF), in Sana'a, Republic of Yemen in the period from January 2021 to June 2022. The data was collected from the patient’s electronic records. Three radiologists with more than 10 years of experience in general US completed the investigations of the patients involved in this study. A linear transducer of 7.5 or 10 MHz of (Samsung Medison, Seoul, Korea) machine was utilized to assess the breast lesions. Real-time, grey-scale, and color Doppler imaging were used to assess each breast lesion. Each lesion was characterized as the following: 1) shape (oval/round, irregular, or lobulated), 2) Border (well-circumscribed, or non-circumscribed), 3) echogenicity (hypoechoic, hyperechoic, isoechoic, or heterogeneous), 4) size, 5) calcification (yes/no), 6) distortion of the surrounding breast tissue (yes/no), 7) nipple retraction (yes/no), 8) skin thickening (yes/no), and 9) the presence of lymph node enlargement in the axilla or near the breast (Figure 1).

US diagnosis for each lesion was done and each lesion was assigned a category [1, 2, 3, 4 (a, b, or c), 5, or 6] according to the fifth edition of the American College of Radiology (ACR) Breast Imaging Reporting and Database System (BI-RADS) US [9]. Patients with doubted, suspicious, or highly suspicious breast malignancy underwent US-guided True-Cut Biopsy (TCB) for a histopathology examination. All biopsy examinations were performed by the same histopathology physician with 14 years of experience. Each biopsy was interpreted and categorized as not diagnostic (B1), benign (B2), lesions with uncertain potential (B3), suspicious of malignancy (B4), and malignant (B5) according to the histopathological B classification [10]. This study involved only the patients who were classified in categories 3, 4 (a, b, or c), 5, or 6 according to ACR BI-RADS categories of the US, and categorized in category B5 by histopathology results. Inclusion criteria: 1) patients who underwent the US and were classified into categories 3, 4, 5, or 6 and confirmed to have breast malignancy by TCB and histopathology results. Exclusion criteria: 1) patients with no clear diagnosis by US, 2) patients who were classified at category 1 or 2 by US, 3) patients who were classified as B1, B2, B3, or B4 by histopathology, and 4) patients with non-malignant/benign lesions of the breast were excluded.

Statistical analysis

The collected data analysis was performed using SPSS (IBM Corp. Released 2017. IBM SPSS Statistics for Windows, Version 25.0. Armonk, NY: IBM Corp). Descriptive statistics were expressed as frequencies and percentages. Continuous statistics were expressed as mean± standard deviation. A cross-tabulation test was performed to identify the correlation between the presence of each US feature (yes/no), and the final US diagnosis (benign/malignant). The strength of association of each feature was measured using the Odds Ratio (OR) which was assumed to be significant when more than one, and a 95% confidence interval (CI). The p-value was assumed to be significant when less than 0.05.

Sensitivity, specificity, positive predictive value (PPV), and negative predictive value (NPV) of each ultra-sonographic diagnostic feature for breast malignancy were calculated using the following equations: sensitivity= [A/(A+C)] × 100, specificity= [D/(B+D) × 100, PPV= A/(A+B)] × 100, and NPV= [D/(C+D)] × 100. While, A is the true positive, B is the false positive, C is the false negative, and D is the true negative [11].

## Results

In total, 326 female patients with breast malignancy were included in this study. The mean age was 45.36 ±12.16 years (range, 17-90 years). Breast malignancies were 49.7% (162) and 50.3% (164) in the right and left breast respectively.

Cross-tabulation test between each ultra-sonographic diagnostic feature and the final US diagnosis shows a significant association between the irregular shape of the lesion and malignancy (p < 0.001, OR = 7.162, CI 2.726-18.814), non-circumscribed margins (p < 0.001, OR=9.031, CI 3.200-25.489), tissue distortion (p < 0.001, OR=18.095, CI 5.944-55.091), nipple retraction (p = 0.002, OR = 7.475, CI 1.722-32.453), skin thickening (p < 0.001, OR = 15.449, CI 2.056-116.117), lymph node enlargement (p < 0.001, OR = 5.705, CI 2.332-13.960). The test shows that hypoechogenicity is not significantly associated with malignancy (p = 0.383, OR = 1.966, CI 0.419-9.229), (Table 1).

**Table 1:** Cross-tabulation test between each ultrasonographic diagnostic feature and the final US diagnosis.

Variable	Variable	Categories	Yes	No	Total	Odds ratio	95% confidence interval		P-value	
							Lower	Upper		
			No. (%)	No. (%)	No. (%)					
Shape	Oval/Round	Malignant	8 (66.7)	295 (93.9)	303 (92.9)	0.710	0.475	1.060	<0.001	
		Benign	4 (33.3)	19 (6.1)	23 (7.1)	5.509	2.215	13.701		
		Total	12	314	326 (100)	0.129	0.036	0.466		
	Irregular	Malignant	282 (94.9)	21 (72.4)	303 (92.9)	1.311	1.046	1.644	<0.001	
		Benign	15 (5.1)	8 (27.6)	23 (7.1)	0.183	0.085	0.395		
		Total	297	29	326 (100)	7.162	2.726	18.814		
	Lobulated	Malignant	17 (81)	286 (93.8)	303 (92.9)	0.863	0.700	1.064	0.027	
		Benign	4 (19)	19 (6.2)	23 (7.1)	3.058	1.144	8.174		
		Total	21	305	326 (100)	0.282	0.086	0.923		
Margins	Well-circumscribed	Malignant	14 (66.7)	289 (94.8%)	303 (92.9)	0.704	0.519	0.953	<0.001	
		Benign	7 (33.3)	16 (5.2)	23 (7.1)	6.354	2.941	13.728		
		Total	21	305	326 (100)	0.111	0.039	0.313		
	Not circumscribed	Malignant	289 (94.8)	14 (66.7)	303 (92.9)	1.421	1.049	1.925	<0.001	
		Benign	16 (5.2)	7 (33.3)	23 (7.1)	0.157	0.073	0.340		
		Total	305	21	326 (100)	9.031	3.200	25.489		
Echogenicity	Hypo echogenicity	Malignant	289 (93.2)	14 (87.5)	303 (92.9%)	1.065	0.883	1.285	0.383	
		Benign	21 (6.8)	2 (12.5)	23 (7.1)	0.542	0.139	2.113		
		Total	310	16	303 (92.9)	1.966	0.419	9.229		
	Heterogeneous echogenicity	Malignant	10 (90.9)	293 (93)	303 (92.9)	0.977	0.809	1.181	0.789	
		Benign	1 (9.1)	22 (7)	23 (7.1)	1.302	0.192	8.805		
		Total	11	315	326 (100)	0.751	0.092	6.136		
Tissue characters	Calcification	Malignant	129 (94.2)	174 (92.1)	303 (92.9)	1.023	0.964	1.085	0.465	
		Benign	8 (5.8)	15 (7.9)	23 (7.1)	0.736	0.321	1.686		
		Total	137	189	326 (100)	1.390	0.572	3.378		
	Tissue distortion	Malignant	240 (98.4)	63 (76.8)	303 (92.9)	1.280	1.136	1.443	<0.001	
		Benign	4 (1.6)	9 (23.2)1	23 (7.1)	0.071	0.025	0.202		
		Total	244	82	326 (100)	18.095	5.944	55.091		
	Nipple retraction	Malignant	126 (98.4)	177 (89.4)	303 (92.9)	1.101	1.045	1.161	0.002	
		Benign	2 (1.6)	21 (10.6)	23 (7.1)	0.147	0.035	0.618		
		Total	128	198	326 (100)	7.475	1.722	32.453		
	Skin thickening	Malignant	125 (99.2)	178 (89)	303 (92.9)	1.115	1.059	1.173	<0.001	
		Benign	1 (0.8)	22 (11)	23 (7.1)	0.072	0.010	0.529		
		Total	126	200	326 (100)	15.449	2.056	116.117		
Others	Lymph node enlargement	Malignant	267 (95.4)	36 (78.3)	303 (92.9)	1.218	1.044	1.422	<0.001	
		Benign	13 (4.6)	10 (21.7)	23 (7.1)	0.214	0.100	0.458		
		Total	280	46	326 (100)	5.705	2.332	13.960		

The calculated sensitivity, specificity, positive predictive value (PPV), negative predictive value (NPV), and diagnostic accuracy for each of the US features of breast malignancy are in (Table 2).

**Table 2:** Ultrasound imaging features of breast lesions.

	Ultrasonographic feature	Sensitivity	Specificity	PPV	NPV	Accuracy
1	Irregular shape	93.1	27.58	94.94	34.78	88.95
2	Non-circumscribed	95.37	30.43	94.75	33.33	90.79
3	Hypo echogenicity	95.37	08.69	93.22	12.50	89.26
4	Tissue distortion	79.20	82.60	98.36	23.17	79.44
5	Nipple retraction	41.58	91.30	98.43	10.60	38.65
6	Skin thickening	41.25	95.65	99.21	11.00	38.34
7	Lymph node enlargement	80.11	43.47	95.35	21.73	81.90

**Figure 2:**
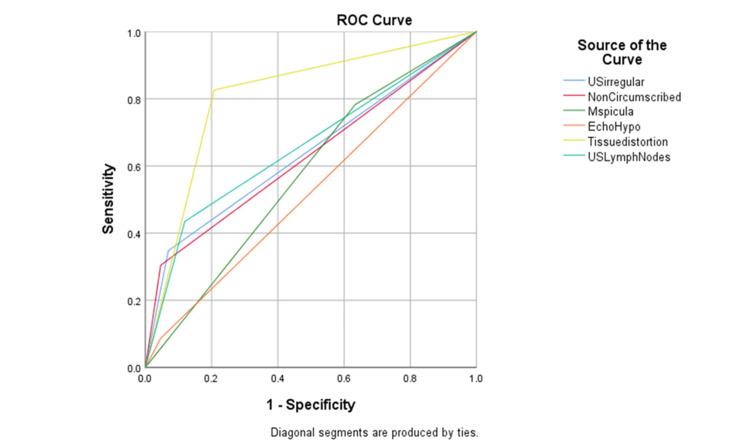
Receiver operating characteristic (ROC) curve shows the area under the curve (AUC) of each feature of BC and revealed that AUC of tissue distortion= 0.809, AUC of lymphadenopathy=0.658, AUC of irregular shape =0.639, AUC of non-circumscribed = 0.629, AUC of speculated margins= 0.574, AUC of hypo-echogenicity=0.520.

The receiver operating characteristic (ROC) curve shows the area under the curve (AUC) for each of the US features of breast malignancy (Figure 2).

## Discussion

US is widely used as a primary tool for the early detection of BC with high sensitivity and specificity. The current study aims to elucidate the sensitivity and predictive value of each US imaging feature for breast malignancy. The US can predict benign breast lesions with high sensitivity and specificity [12]. US can distinguish between benign and malignant solid breast lesions with high accuracy, and 99.5% NPV [13]. In the current study, the breast lesions classified in Breast Imaging Reporting & Data System (BIRADS) 1 and BIRADS2 were excluded because they are almost always benign according to previous studies [8,9].

The shape, margins, internal echotexture, and posterior echo are the most significant features in differentiating benign from malignant breast lesions in tumors > 2 cm. However, the margin of the breast lesion is the only significant feature in differentiating malignant breast lesions ≤1 cm. [14]. In the current study, we studied the shape, margins, internal echotexture, and surrounding tissue features in histopathological proven malignant breast lesions. We found that irregular shape, non-circumscribed margins, and hypo-echogenicity of breast lesions are highly sensitive features for diagnosis of BC with very high sensitivity and PPV. However, the specificity and NPV of these features were not specific features related to malignancy with weak NPV to be used to exclude BC. This result is explained by the published research of Kim et al. who reported that irregular hypoechoic breast lesions are usually considered suspicious, however, many benign breast lesions can present as irregular, and hypoechoic masses that can mimic BC. 1) iatrogenic or trauma-related breast lesions such as fat necrosis, fibrotic scar, or foreign body reaction, 2) inflammatory such as abscess, idiopathic granulomatous lobular mastitis, and diabetic mastopathy, 3) proliferative diseases such as sclerosing adenitis, fibrocystic changes, and apocrine metaplasia, 4) benign breast neoplasms such as intraductal papilloma, fibroadenoma, and tubular adenoma [15]. 

In another study, Marino et al. reported that the detection of enlarged metastatic axillary lymph nodes affects the management of patients with BC in staging, treatment, and prognosis, and ultrasound imaging is the imaging modality of choice for evaluating axillary lymph nodes [16]. Our results show that the presence of enlarged lymph nodes in conjunction with breast mass is highly valuable in diagnosing BC. However, enlarged lymph nodes are not a specific feature of BC and have low NPV. This is explained by Dialani et al. who reported that enlarged axillary lymph nodes may be seen in benign as in malignant breast lesions in addition to other entities such as reactive hyperplasia, HIV/immunocompromised patients, granulomatous diseases, and malignancies in other tissues [17].

Our study shows that distortion of the breast tissue is a highly significant feature with high sensitivity and specificity for diagnosing breast malignancy, however, it has low NPV. The low NPV of tissue distortion is explained by Gaur et al. who reported that tissue distortion can be seen in US imaging or other imaging modalities in benign breast lesions such as a radial scar, sclerosing adenosis, fat necrosis, breast fibromatosis, and even in post-procedural changes [18]. Kim et al. reported that only 35% of breast cases with architectural distortion on ultrasound imaging were BC, and 35% were mild-risk lesions [19].

In comparison to other imaging modalities, Ultrasound imaging can also diagnose small malignant lesions (≤ 1cm) which may be occult lesions on mammography, especially on dense breast parenchyma. The irregular shape and not circumscribed margins are the strong predictive signs of malignancy [20]. Moreover, Mahoney et al. reported that magnetic resonance imaging (MRI) morphological features of malignancy have the highest PPV for irregular shape, and irregular and spiculated margins of the breast lesions [21]. 

Limitations; This study was limited in the invalidity of detailed features of the margins of the lesions (indistinct, angular, or micro lobulated) in most of the preserved reports of the involved patients, so that, we involved only the available features in this study. The type of calcification was not determined into microcalcification or other types in the available reports of the US and the nature of this technique cannot detect calcification in all patients.

Future studies about the efficacy of US imaging in determining the presence of microcalcification and other types of calcifications in comparison with Mammography are recommended.

## Conclusions

US imaging features of malignancy have a high sensitivity in the detection of BC. However, the specificity of breast US imaging features is much lower because of the overlapping features in benign and malignant breast lesions. Breast lesions with an irregular shape, not circumscribed irregular or spiculated margins, hypoechogenicity, tissue distortion, and those with lymphadenopathy have the highest likelihood of malignancy despite the low specificity. US is a highly valuable, safe, and cheap, imaging modality with high diagnostic accuracy for BC.
